# Phylogenetic analysis of the ATP-binding cassette proteins suggests a new ABC protein subfamily J in *Aedes aegypti* (Diptera: Culicidae)

**DOI:** 10.1186/s12864-020-06873-8

**Published:** 2020-07-06

**Authors:** Janaina Figueira-Mansur, Carlos G. Schrago, Tiago S. Salles, Evelyn S. L. Alvarenga, Brenda M. Vasconcellos, Ana Claudia A. Melo, Monica F. Moreira

**Affiliations:** 1 Laboratório de Bioquímica e Biologia Molecular de Vetores, Departamento de Bioquímica, Instituto de Química, Universidade Federal do Rio de Janeiro, Rio de Janeiro, RJ 21941-909 Brazil; 2 Departamento de Genética, Instituto de Biologia, Universidade Federal do Rio de Janeiro, Rio de Janeiro, RJ 21941-617 Brazil; 3 Instituto Nacional de Ciência e Tecnologia em Entomologia Molecular, Rio de Janeiro, RJ Brazil

**Keywords:** *Aedes aegypti*, MutS, RAD50 and SMC proteins, MDR phenotype, ABC protein classification, ABC protein subfamily J

## Abstract

**Background:**

We performed an in-depth analysis of the ABC gene family in *Aedes aegypti* (Diptera: Culicidae), which is an important vector species of arthropod-borne viral infections such as chikungunya, dengue, and Zika. Despite its importance, previous studies of the Arthropod ABC family have not focused on this species. Reports of insecticide resistance among pests and vectors indicate that some of these ATP-dependent efflux pumps are involved in compound traffic and multidrug resistance phenotypes.

**Results:**

We identified 53 classic complete ABC proteins annotated in the *A. aegypti* genome. A phylogenetic analysis of *Aedes aegypti* ABC proteins was carried out to assign the novel proteins to the ABC subfamilies. We also determined 9 full-length sequences of DNA repair (MutS, RAD50) and structural maintenance of chromosome (SMC) proteins that contain the ABC signature.

**Conclusions:**

After inclusion of the putative ABC proteins into the evolutionary tree of the gene family, we classified *A. aegypti* ABC proteins into the established subfamilies (A to H), but the phylogenetic positioning of MutS, RAD50 and SMC proteins among ABC subfamilies—as well as the highly supported grouping of RAD50 and SMC—prompted us to name a new J subfamily of *A. aegypti* ABC proteins.

## Background

The ATP-binding cassette (ABC) transporters constitute a diverse gene family consisting of proteins found in all cellular organisms and participating in several different biological pathways [1]. Among these processes, the ABC transporters are mostly involved in extra and intracellular trans membrane ATP energy driven traffic of molecules such as lipids, amino acids, hormones and xenobiotics [2, 3].

Members of this family are characterized by two trans membrane domains (TMD) and two nucleotide-binding domains (NBD) characterized by conserved motifs: Walker A, Walker B, ABC signature (LSGGQ-motif), Q loop, and H loop [1, 4]. The TMD domains of the ABC-transporters are composed of five to ten membrane-spanning regions that are involved in substrate translocation. The four domains (two TMD and two NBD) of a functional ABC transporter might be present in a single protein (full transporter) or in dimers of separate proteins that have at least one TMD and one NBD each (half transporter) [3, 5].

The traditional classification is based on sequence similarity and arranged the ABC protein diversity into eight subfamilies (A- H) [6]. The ABCE and ABCF subfamilies are unique among the ABC proteins because they exhibit a pair of linked nucleotide-binding domains while lacking trans membrane domains [3, 6]. The ABCH subfamily was described for protozoa [7] and insects [8, 9], but it has not yet been found in mammals, bacteria, and yeast genomes. Plants, besides presenting eukaryotic ABC subfamilies A to G, exhibit a heterogeneous and extensive group of ABC proteins that bear similarities to the components of prokaryotic multi-subunit ABC transporters. This group was named subfamily I and includes NBD and TMD domains and homologues of soluble cytosolic proteins that interact with NBDs as well as putative substrate-binding proteins similar to the periplasmic binding proteins [10].

Three other groups of proteins not assigned to the subfamilies mentioned above exhibit ABC transporter domains: (1) the MutS proteins that are responsible for DNA mismatch repair and maintenance of genomic stability [11, 12]; (2) the structural maintenance of chromosome proteins (SMC), which are mostly responsible for chromosome condensation and sister chromatid cohesion [13], and (3) the Rad 50 proteins that also function on DNA repair [8, 9, 14].

Although MutS, SMC, and Rad50 proteins show ABC protein characteristics, they have not yet been included in the standard ABC classification for humans, arthropods, and the *Caenorhabditis elegans* nematode [8, 9, 15, 16]. Nonetheless, in the complete inventory of ABC proteins of the *Arabidopsis thaliana* plant, SMC proteins were proposed as a new ABC protein subfamily [17].

Some ABC proteins have been associated with multidrug resistance (MDR) phenotype in a variety of organisms. This phenotype is associated with the overexpression of P-glycoproteins (P-gp/MDR/ABCB1), the multidrug resistance protein (MDR/ABCC), and the breast cancer resistance protein (BCRP/ABCG2) [5, 18, 19]. These act as efflux pumps that result in resistance to chemotherapeutics, antibiotics, and antiretroviral drugs [20, 21].

One important control mechanism of vector-borne diseases is vector control, which relies mainly on insecticide treatments of vector populations. In these populations, the insecticide-resistant phenotype arises due to the selection of genetically resistant individuals that exhibit higher fitness under special conditions [22, 23]. Multiple insecticide resistance can be separated into two main categories: cross-resistance—when a single mechanism confers resistance to a range of different insecticides; and multiple resistance—when several coexisting defense mechanisms act in the same organism [24, 25]. The involvement of ABC transporters in insecticide resistance and transport is poorly documented, but an increasing number of studies have shown that ABC transporters have been linked to insecticide and nicotine transport [26–28] and insect resistance to *Bacillus thuringiensis* toxins and pyrethroids [29, 30]. The high expression of P-gp in insecticide resistant pests such as *Heliothis virescens* and *Helicoverpa armigera* has been suggested to be a mechanism of resistance [31, 32].

Recent surveys of the ABC gene family in arthropods included the fruit fly *Drosophila melanogaster*, the mosquito *Anopheles gambiae*, the beetle *Tribolium castaneum*, the honey bee *Apis mellifera*, the silkmoth *Bombyx mori*, the water flea *Daphia pulex*, and the spider mite *Tetranychus urticae* [16]. Analyses focusing on crustaceans such as the sea lice *Caligus rogercresseyi* [33] and *Lepeophtheirus salmonis* [34] were also carried out. These studies left out the *A. aegypti* mosquito, which is an important vector species of arthropod-borne viral infections such as chikungunya, dengue, and Zika diseases [35]. In 2016, Lu et al. [36] conducted a comparative analysis of the ABC transporter family in three mosquito species (*Anopheles gambiae*, *Aedes aegypti*, and *Culex pipiens quinquefasciatus*) and found 55, 69, and 70 ABC genes, respectively. The search for *Aedes aegypti* ABC proteins, however, was carried out within a limited evolutionary range because only mosquito sequences were analyzed.

In this study, we surveyed the *Aedes aegypti* genome in a broader evolutionary spectrum, employing human and *Drosophila* ABC transporters as queries. By including all the putative proteins that exhibit the ABC domain into a phylogenetic analysis, we showed that SMC, Rad 50, and MutS proteins were part of the main ABC gene family diversification, which justifies the proposition of a new subfamily of the ABC proteins.

## Results

**Fig. 1:**
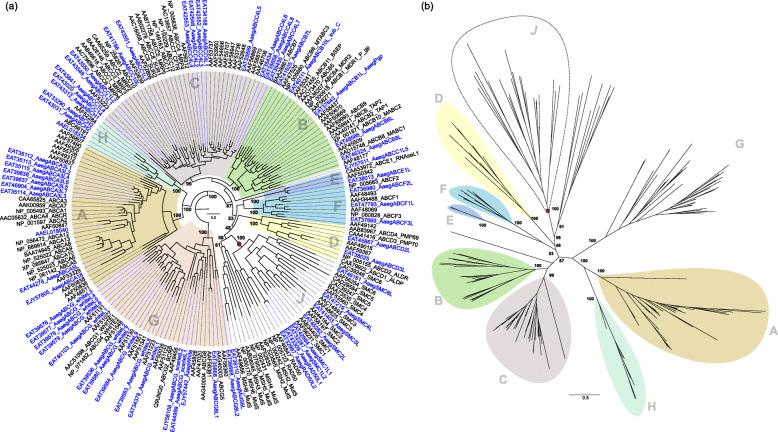
**a** Maximum likelihood phylogeny of the ABC gene family including SMC, Rad50 and MutS genes. ABC subfamilies are shown with the new mosquito sequences highlighted in blue. **b** Numbers at branches indicate statistical support (ultra-fast bootstrap) for each subfamily A-J

The BLASTp search on the *A. aegypti* genome retrieved 62 complete proteins that were identified as ABC transporters when submitted to the NCBI Conserved Domain Database. The ABC gene family phylogeny recovered subfamilies A-H with significant statistical support (Fig. 1). The sizes of gene subfamilies varied significantly with subfamilies A-C and G consisting of the larger groups. Sister group associations between ABC subfamilies were less resolved. The single exception was the clade with subfamilies ABCA and ABCH that were grouped with maximum statistical support. In all ABC subfamilies, *A. aegypti* proteins had a tendency to be positioned among human and *Drosophila* sequences suggesting that the duplication events that gave rise to current ABC diversity took place before the evolution of those lineages. Clusters containing ABC genes exclusively from *A. aegypti* were found in subfamilies ABCA, ABCC, and ABCG. These clusters indicate mosquito-specific duplication events.

**Table 1:** Classification of ABC proteins subfamilies in *Homo sapiens* and *Drosophila melanogaster*

***Homo sapiens***			***Drosophila melanogaster***		
Sub-family	Seq ID	GenBank accession	Sub-family	Seq ID	GenBank accession
A	ABCA1	NP_005493	A		AAF50836
A	ABCA2	NP_001597	A		AAF50837
A	ABCA3	CAA65825	A		AAF50838
A	ABCA4_ABCR	AAC05632	A		AAF50847
A	ABCA5	NP_061142	A		AAF53329
A	ABCA6	NP_525023	A		AAF55726
A	ABCA7	AAK00959	A		AAF57490
A	ABCA8	BAA74845	B		AAF45509
A	ABCA9	NP_525022	B		AAF47525
A	ABCA10	XP_085647	B		AAF48177
A	ABCA12	NP_056472	B		AAF50669
A	ABCA13	NP_689914	B		AAF50670
B	ABCB1_MDR1_P_gp	NP_000918	B		AAF53736
B	ABCB2_TAP1	CAA40741	B		AAF53737
B	ABCB_TAP2	AAA59841	B		AAF55241
B	ABCB4_MDR3	AAA36207	B		AAF58271
B	ABCB5	AAO73470	B		AAF58437
B	ABCB6_MTABC3	NP_005680	C		AAF46706
B	ABCB7	BAA28861	C		AAF52639
B	ABCB8_MABC1	AAD15748	C		AAF52648
B	ABCB9	AAF89993	C		AAF52866
B	ABCB10_MABC2	XP_001871	C		AAF53223
B	ABCB11_BSEP	AAC77455	C		AAF53950
C	ABCC1_MRP1	AAB46616	C		AAF54656
C	ABCC2_MRP2	CAA65259	C		AAF55707
C	ABCC3_MRP3	BAA28146	C		AAF56312
C	ABCC4_MRP4	NP_005836	C		AAF56869
C	ABCC5_MRP5	AAB71758	C		AAF56870
C	ABCC6_MRP6	AAC79696	C		AAF58947
C	ABCC7_CFTR	AAC13657	D		AAF49018
C	ABCC8_SUR1	AAB02278	D		AAF59367
C	ABCC9_SUR2	AAC16058	E		AAF50342
C	ABCC10	NP_258261	F		AAF48069
C	ABCC11	NP_149163	F		AAF48493
C	ABCC12	NP_150229	F		AAF49142
D	ABCD1_ALDP	CAA79922	G		AAF45826
D	ABCD2_ALDR	NP_005155	G		AAF47020
D	ABCD3_PMP70	CAA41416	G		AAF49455
D	ABCD4_PMP69	AAB83967	G		AAF50035
E	ABCE1_RNAseL1	CAA53972	G		AAF51027
F	ABCF1	AAH34488	G		AAF51122
F	ABCF2	NP_005683	G		AAF51130
F	ABCF3	NP_060828	G		AAF51131
G	ABCG1_WHITE1	AAC51098	G		AAF51223
G	ABCG2_BCRP	Q9UNQ0	G		AAF51341
G	ABCG4_WHITE2	NP_071452	G		AAF51548
G	ABCG5	AAG40003	G		AAF51551
G	ABCG8	AAG40004	G		AAF52835
J	SMC1	AAB34405	G		AAF56360
J	SMC4	BAA73535	G		AAF56361
J	SMC3	AAC14893	H		AAF52284
J	SMC2	AAI44164	H		AAF56807
J	RAD50	NP_005723	H		ABC66191
J	SMC5	CAC39247	J	SMC1	AAF56231
J	SMC6	CAC39248	J	SMC4	AAF53560
J	MSH4_MutS	NP_002431	J	SMC2	AAF58197
J	MSH3_MutS	AAB06045	J	RAD50	AAF46847
J	MSH5_MutS	NP_079535	J	SMC5	CAD29584
J	MSH2_MutS	NP_000242	J	SMC6	AAF56254
J	MSH6_MutS	NP_000170	J	MSH2_MutS	NP_523565
			J	MSH6_MutS	AAF49656
			J	SMC3	AAF48625

The variation of the rate of evolution within each ABC subfamily as measured by the heterogeneity of the distance between the common ancestor of all members of the subfamily and the tips was higher in subfamily ABCA. In this subfamily, an interesting pattern of rate increase along lineages was observed (Fig. 1). As expected, deeper nodes exhibited lower statistical support demonstrating that the evolutionary relationships between these subfamilies were not fully resolved. Surprisingly, root placement using the minimal ancestor deviation (MAD) method suggested that subfamily ABCG is a sister to the remaining ABC transporters including the clades consisting of SMC and Rad50 proteins as well as the MutS proteins that were positioned as a sister to subfamily ABCD (Fig. 1).

## Discussion

To investigate ABC transporters in the *A. aegypti* genome within a broader evolutionary context, we identified *A. aegypti* ABC homologs employing human and *D. melanogaster* as queries (Table 1). We also identified the conserved domains of all the putative *A. aegypti* ABC transporters to investigate the assignment of the putative proteins to the described subfamilies of these transporters. We identified ten members of the *A. aegypti* ABCA subfamily (Fig. 1 and Table 2). This subfamily contains longer proteins that ranged from 1419 to 1673 amino acid residues. Nine of these members have the topology of full transporters with two NBDs and two TMDS (Table 2). The *A. aegypti* ABCA subfamily was encoded by genes organized in tandem indicating specific gene duplication events (Table 2). Four members of this cluster have genes organized in tandem in the supercontig 1.726, two members belong to the supercontig 1.321, and four belong to other supercontigs (Table 2). The roles of arthropod ABCA members are unclear [16], but this subfamily has been described as involved with lipid transport in mammals [37].

**Table 2:** Characterization of the 62 *A. aegypti* ABC proteins

Sub-family	Name	VectorBase (accession number)	Size (amino acids)	Predicted topology	Location (gene)	Orientation (gene)
**A**						
	AaegABCA3L1	AAEL012702-PA	1669	TMD1-NBD1-TMD2-NBD2	1.726: 372101–377,726	+
	AaegABCA3L2	AAEL012700-PA	1648	TMD1-NBD1-TMD2-NBD2	1.726: 388899–394,375	+
	AaegABCA3L3	AAEL012701-PA	1622	TMD1-NBD1-TMD2-NBD2	1.726: 409854–439,050	+
	AaegABCA3L4	AAEL012698-PA	1652	TMD1-NBD1-TMD2-NBD2	1.726: 450626–459,977	+
	AaegABCA3L5	AAEL008388-PA	1666	TMD1-NBD1-TMD2-NBD2	1.321: 644618–664,804	–
	AaegABCA3L6	AAEL008384-PA	1660	TMD1-NBD1-TMD2-NBD2	1.321: 675803–697,600	–
	AaegABCA3L7	AAEL001938-PA	1673	TMD1-NBD1-TMD2-NBD2	1.46: 792516–818,527	–
	AaegABCA5L	AAEL004331-PA	1419	TMD1-NBD1-TMD2-NBD2	1.115: 240545–271,476	+
	AaegABCA5L	AAEL018040-PA	1987	TMD1-NBD1-TMD2-NBD2	3.322: 613800–714,818	–
	AaegABCA18	AAEL017572-PA	347	NBD	1.176: 1628836–1,629,879	–
**B**						
	AaegABCB1L/AaegP-gp	AAEL010379-PA	1307	TMD1-NBD1-TMD2-NBD2	1.474: 313030–327,570	+
	AaegABCB6L	AAEL000434-PA	693	TMD-NBD	1.8: 3711414–3,730,662	+
	AaegABCB7L	AAEL006717-PA	734	TMD-NBD	1.219: 178589–203,717	–
	AaegABCB8L	AAEL002468-PA	703	TMD-NBD	1.58: 1203051–1,224,141	–
	AaegABCB10L	AAEL008134-PA	848	TMD-NBD	1.302: 73729–107,503	+
**C**						
	AaegABCC1L1	AAEL005026-PA	1384	TMD0-TMD1-NBD1-TMD2-NBD2	1.139: 1168407–1,184,363	+
	AaegABCC1L2	AAEL005045-PA	1514	TMD0-TMD1-NBD1-TMD2-NBD2	1.139: 1184563–1,195,380	–
	AaegABCC1L3	AAEL005030-PA	1396	TMD0-TMD1-NBD1-TMD2-NBD2	1.139: 1233513–1,252,972	–
	AaegABCC1L4	AAEL004743-PA	1089	TMD0-TMD1-NBD1	1.129: 994901–1,030,978	+
	AaegABCC1L5	AAEL017209-PA	903	TMD0 -TMD1-NBD1	1.107: 820177–825,969	–
	AaegABCC4L1	AAEL013567-PA	1311	TMD1-NBD1-TMD2-NBD2	1.871: 281423–317,150	+
	AaegABCC4L2	AAEL005918-PA	1312	TMD1-NBD1-TMD2-NBD2	1.180: 664096–681,744	–
	AaegABCC4L3	AAEL005937-PA	1300	TMD1-NBD1-TMD2-NBD2	1.180: 724473–765,746	+
	AaegABCC4L4	AAEL005929-PA	1413	TMD1-NBD1-TMD2-NBD2	1.180: 786121–801,780	+
	AaegABCC4L5	AAEL013834-PA	1235	TMD1-NBD1-TMD2-NBD2	1.936: 291553–353,031	–
	AaegABCC4L6	AAEL012395-PA	1357	TMD1-NBD1-TMD2-NBD2	1.688: 67831–72,390	–
	AaegABCC4L7	AAEL012386-PA	1351	TMD1-NBD1-TMD2-NBD2	1.688: 87463–91,714	+
	AaegABCC4L8	AAEL012192-PA	1345	TMD1-NBD1-TMD2-NBD2	1.664: 660781–670,973	–
	AaegABCC10L	AAEL006622-PA	1540	TMD0-TMD1-NBD1-TMD2-NBD2	1.213: 838086–915,438	+
	AaegABCC14	AAEL005499-PA	1382	TMD1-NBD1-TMD2-NBD2	1.160: 1362499–1,398,139	–
**D**						
	AaegABCD2L	AAEL002913-PA	659	TMD-NBD	1.71: 1617561–1,676,168	+
	AaegABCD3L	AAEL010047-PA	753	TMD-NBD	1.449: 843528–895,566	+
**E**						
	AaegABCE1L	AAEL010059-PA	609	NBD1-NBD2	1.450: 713084–727,146	+
**F**						
	AaegABCF1L	AAEL001101-PA	894	NBD1-NBD2	1.23: 2941514–2,961,984	–
	AaegABCF2L	AAEL010977-PA	602	NBD1-NBD2	1.529: 122943–143,748	–
	AaegABCF3L	AAEL010359-PA	609	NBD1-NBD2	1.450: 713084–727,146	+
**G**						
	AaegWhite*	AAEL016999-PA	692	NBD-TMD	1.71: 816675–879,820	–
	AaegABCG/whiteL1	AAEL008138-PA	773	NBD-TMD	1.303: 412767–432,830	+
	AaegABCG/whiteL2	AAEL008672-PA	689	NBD-TMD	1.340: 378892–469,513	+
	AaegABCG/whiteL3	AAEL008624-PA	593	NBD-TMD	1.337: 23491–61,022	–
	AaegABCG/whiteL4	AAEL008632-PA	607	NBD-TMD	1.337: 68512–71,099	–
	AaegABCG/whiteL5	AAEL008628-PA	571	NBD-TMD	1.337: 85665–99,799	–
	AaegABCG/whiteL6	AAEL008625-PA	606	NBD-TMD	1.337: 119628–131,014	+
	AaegABCG/whiteL7	AAEL008629-PA	723	NBD-TMD	1.337: 131034–224,797	–
	AaegABCG/whiteL8	AAEL008631-PA	759	NBD-TMD	1.337: 276979–394,542	–
	AaegABCG/whiteL9	AAEL008635-PA	676	NBD-TMD	1.337: 470559–525,334	–
	AaegABCG/whiteL10	AAEL013372-PA	599	NBD-TMD	1.830: 256110–301,680	+
	AaegABCG/scarletL1	AAEL003703-PA	616	NBD-TMD	1.94: 984346–994,398	–
	AaegABCG/scarletL2	AAEL017106-PA	689	NBD-TMD	1.1174: 142758–145,023	–
	AaegABCG8L1	AAEL012170-PA	275	NBD	1.662: 13565–20,660	+
	AaegABCG8L2	AAEL011265-PA	787	NBD-TMD	1.561: 434947–481,728	+
**H**						
	AaegABCH1	AAEL005249-PA	872	NBD-TMD	1.147: 1132338–1,176,150	+
	AaegABCH2	AAEL005491-PA	783	NBD-TMD	1.159: 901905–920,193	+
	AaegABCH3	AAEL014428-PA	727	NBD-TMD	1.1111: 126773–184,623	+
	AaegABCH4L5	AAEL018334-PA	814	NBD-TMD	1.181:178199–420,930	–
**J**						
	AaegSMC1L1	AAEL005802-PA	1227	NBD	1.175: 1418947–1,437,720	–
	AaegSMC1L2	AAEL015592-PA	594	NBD	1.3565: 4371–6283	–
	AaegSMC2L	AAEL003449-PA	1182	NBD	1.86: 847376–874,751	–
	AaegSMC3L	AAEL006937-PA	1201	NBD	1.229: 536438–586,285	–
	AaegSMC4L	AAEL001655-PA	1347	NBD	1.38: 1385452–1,432,324	+
	AaegSMC6L	AAEL002581-PA	1107	NBD	1.61: 165187–198,927	+
	AaegRAD50L1	AAEL005245-PA	1034	NBD	1.147: 747831–755,196	–
	AaegRAD50L2	AAEL014748-PA	1051	NBD	1.1248: 101280–115,288	–
	AaegMutS6L	AAEL011780-PA	1130	NBD	1.612: 217022–240,932	+

Five sequences retrieved from the *A. aegypti* genome were assigned to the ABCB subfamily (Fig. 1, Table 2). This subfamily is composed of putative homologs of the human P-glycoprotein, which plays key physiological roles such as the excretion of toxic compounds and the multidrug resistance phenotype [3, 26, 27, 37, 38]. The identified *A. aegypti* ABCB proteins are intimately related to the human mitochondrial transporters HsABCB6, HsABCB7, HsABCB8, and HsABCB10 leading us to suppose that these proteins have a similar role associated with the iron metabolism and the transport of Fe/S protein precursors from the mitochondria to the cytoplasm [37, 39]. We also note that one *D. melanogaster* protein classified as belonging to the ABCB (CG31792_B) subfamily was recovered in the ABCC clade. This may be due to misclassification or to recent duplication and functional change. In either case, this protein should be further investigated.

One of the most diverse subfamilies identified in the mosquito genome was the ABCC with 15 members—all full transporters (Table 2). This subfamily presents a high diversity of sequences as well as functional roles when compared with the human ABCC proteins. These functions are related to ion transport, cell surface receptors, toxin secretion, and multidrug resistance [38]. A sub-clade containing all the MRP from humans and *D. melanogaster* was recovered including four *A. aegypti* proteins (AaegABCC1L1, AaegABCC1L2, AaegABCC1L4, and AaegABCC1L5) suggesting that these proteins might also be responsible for protection against xenobiotics [40] and for the MDR phenotype [38, 41].

The ABCD and ABCE subfamilies were the least diverse of the groups identified in humans—the former is known to appear as half transporters forming homo or heterodimers in peroxisomes acting in lipid transport [3, 39, 42]. The ABCD subfamily has two members and the ABCE subfamily has only one protein described for most eukaryotes (Table 2) with the exception of *A. thaliana* [17]. This was consistent with the findings of a single ABCE gene in the *A. aegypti* genome. These proteins lack the TMD and were first described as the RNAseL protein participating in ribosome biogenesis and protein translation [37–39, 43–46]. Like ABCE proteins, the ABCF subfamily also lacks the TMD and is involved in the ribosome complex formation and activation [46–48]; only three of these proteins were found in the mosquito genome in our analysis.

Although only five members of the ABCG proteins were described in humans [3, 37], 15 proteins belonging to this group were identified for *A. aegypti* (Table 2). This number is greater than the 11 genes previously identified in *An. gambiae* [9]. This excessive number of ABCG proteins in *A. aegypti* mosquito is likely due to a series of duplication events that is supported by the tandem organization observed in the supercontig 1.337 of the *A. aegypti* genome (Table 2). In *D. melanogaster*, the white gene is the most studied gene from the ABCG subfamily, and the product of this gene can form dimers with the scarlet and brown proteins (scarlet and brown genes, respectively). These dimers are transporters of eye pigment precursors in *D. melanogaster* [49, 50]. Only one ortholog of the white and scarlet proteins was found in the *A. aegypti* genome but no ortholog of the brown protein was found. In humans, ABCG5 and ABCG8 are glycoproteins that also form obligate heterodimers. These are useful to limit the absorption of plant sterols and cholesterol from the diet and promote secretion of plant sterols and cholesterol from liver cells into the bile. Based on their head-to-head orientation and clear orthologous relationships with human ABCG5 and ABCG8, these arthropod ABCGs probably have a similar role as their human orthologues [37].

The ABCH subfamily was exclusively found in insects with no reports in mammals, plants, or yeast [9, 37]. Here, four members of the ABCH subfamily were identified in the *A. aegypti* genome (Fig. 1 and Table 2). This included the sequence AAEL018334, which has been previously assigned to ABCG subfamily. Although these are proteins with unknown function, topological similarities with the ABCG proteins have suggested that the ABCH might be involved in sterol transport and multidrug resistance [51, 52].

Insect P-glycoproteins and multidrug-resistance associated proteins are frequently associated with pesticide resistance as reported in *Heliothis virescens* and *Helicoverpa armigera* [30, 31] and insecticide transport. The expression of *A. aegypti* P-gp (AAEL010379) increases eightfold in the temephos-treated larvae, and silencing of this gene expression significantly increases temephos toxicity [27]. These findings suggested that ABC transport, which consists of ATP-dependent efflux pumps, might be involved with compound traffic and multidrug resistance phenotypes. New insights into insecticide efflux, ATP-dependent efflux pump inhibitors, and/or RNAi associated with pesticides will potentially assist in the development of control strategies for important vectors of infectious diseases like *A. aegypti*.

Rad50 shares topological and sequence features with SMC proteins [52]. Notably, Rad50 has a relatively well-conserved LSGG motif compared to the classic ABC proteins. Moreover, it has an extensive coiled region that facilities dimerization of large molecules restoring the close proximity of the Walker A and B motifs for nucleotide binding [53]. SMCs have more degenerated versions of this signature motif and contain minimal Walker A and B motifs (Supplemental material 1) [54]. Finally, perhaps a distant lineage but still within the ABC diversification [55], are the DNA repair enzymes such as MutS [56].

SMC proteins formed a highly supported clade with the Rad50 proteins. These proteins form dimers and have a conserved mechanism of conformational change observed in the classic ABC proteins. The ATP binding and NBD dimerization promote changes in the substrate-binding domains that are important for the function of the ABC-type ATPases. The substrate-binding domains of the SMC and Rad50 proteins are located in similar positions as the classic ABC proteins [52]. The ABC proteins subfamilies are grouped together based on sequence similarity and proteins belonging to the same subfamily usually have similar functions. Our results showed that ABC subfamilies were always strongly recovered in the gene family phylogeny and that the sequences of SMC and Rad50 proteins formed a well-supported clade (100 bootstrap support), sister to MutS proteins, and ABC transporters excluding ABCG. Functional similarities are also observed within the groups.

We know the following: (i) SMC and Rad50 proteins exhibit similar functions on DNA repair and chromosomal maintenance [8, 9, 11, 12, 14], (ii) they form a strongly supported clade with ABC transporters phylogeny, and (iii) they exhibit the structural and sequence characteristics of ABC proteins. Thus, we propose these proteins be included in the ABC gene family with the creation of a new subfamily called J (Fig. 1; Table 2) that includes ABC proteins involved in DNA repair and structural maintenance of the chromosomes.

## Conclusions

In summary, we found 53 classic complete ABC proteins annotated in the *A. aegypti* genome that were classified in traditional ABC subfamilies (A-H) as reported in other species. We also found 9 sequences of the Rad, MutS, and SMC in the *Aedes* genome database that clustered with human and *D. melanogaster* orthologs in the same clade. Considering other similarities observed between these enzymes and the classic ABC proteins, we propose these proteins be included in the ABC gene family followed by creation of a new subfamily called J that includes ABC enzymes involved in DNA repair and the structural maintenance of the chromosome.

## Methods

### Sequence sampling and alignment

We selected protein sequences of ABC protein subfamilies from humans (46 sequences) and *Drosophila melanogaster* (50 sequences) including sequences from SMC, Rad50, and MutS genes ensuring a broad evolutionary diversity. These sequences were used as query for BLAST searches of *A. aegypti* ABC proteins. To identify *A. aegypti* ABC proteins, human and *Drosophila* sequences were used as queries to search sequences on the mosquito genome (VectorBase) and on the NCBI protein database using BLASTp [57]. Sequences resulting from these searches that had more than 30% identity were considered as homologous (Supplemental material 2).

All putative homologous sequences were submitted to the NCBI Conserved Domain Database [58, 59] to confirm the presence of the ATP-binding cassette domain. Datasets were aligned using the MUSCLE software [60] and then pruned for removal of regions with high frequency of indels using TrimAL using the “gappyout” command (Supplemental material 3) [61].

### Inference of ABC gene genealogy

The maximum likelihood (ML) tree topology was inferred with the IqTree 1.6 [62] program employing the LG + R10 model of amino acid substitution that was chosen by the Bayesian information criterion. This model uses the LG amino acid replacement matrix [63] coupled with ten relative rate classes to accommodate among-site rate heterogeneity [64]. Branch support was assessed by the ultrafast bootstrap implementation of IqTree using 1000 replicates [65]. IqTree was executed via the command “iqtree -s *infile* -bb 1000”. Because no outgroup was included in our analysis, rooting of the ABC gene genealogy was performed using the minimal ancestor deviation method of Tria at al. [66]. Rooting is necessary for establishing the chronological direction of the ABC gene family evolution (Supplemental material 4).

## Supplementary information

**Additional file 1.** Sequences.txt: Rad50, SMC and MutS sequences highlighted Walker A and Walker B motifs and ABC signature.

**Additional file 2.** ABC.txt: Unaligned ABC sequences used in this study.

**Additional file 3.** ABCalin.txt: Aligned and trimmed ABC sequences employed in evolutionary analyses.

**Additional file 4.** ABC.unrooted.txt: Unrooted maximum likelihood phylogenetic tree of the ABC gene family.

## Data Availability

The datasets analysed during the current study are available in the GenBank and VectorBase repositories. All accession numbers were listed in Tables 1 and 2.

## Abbreviations

MDR: Multidrug resistance protein
P-gp: P-glycoproteins
BCRP: Breast cancer resistance protein
SMC: Structural maintenance of chromosome proteins
TMD: Trans membrane domains
NBD: Nucleotide-binding domains
NCBI: National Center for Biotechnology Information
MAD: Minimal ancestor deviation
