# Supplementary material for: Phylogenetic analysis of the ATP-binding cassette proteins suggests a new ABC protein subfamily J in Aedes aegypti (Diptera: Culicidae)
Source: BMC Genomics. 2020 Jul 6;21:463. doi: 10.1186/s12864-020-06873-8 (PMC7339416; doi:10.1186/s12864-020-06873-8)
Supplement: Supplementary file 1 — Additional file 1. Sequences.txt: Rad50, SMC and MutS sequences highlighted Walker A and Walker B motifs and ABC signature. [file 12864_2020_6873_MOESM1_ESM.pdf]

Supplementary material: Rad50, SMC and MutS sequence proteins highlight Walker A, Signature and Walker B domains in this order.

>AAEL005802-PA.NBD1
MDSAFLQFIEVDNFKSYRGKVTIGPLKKFSAVIGPNGSGKSNFMDAISFVMGEKTTSLRVRKLNELIHGA
SIGRPVSNRASVMAKFIITDQEGNQTSKSFQRSVIGSSSEYRINGSVVTTNAYLQELEHIGINVKAKNFL
VFQGAVESIAMKNAKERTALFEEISGSGLLKEEYNKLKHEMQMAEEETQFTYQKKKGIAAERKEAKLEKQ
EADRYARLKDEYAEKQIQYQLYRLYHNEKETKRLYEDMESKQQDLTLIEKKKEEADDVLKEKKKEAGKTA
RDLAKNEQEIREVESEMSKKHPMFIKAKEKVAHTKKKLDSAMKTLEQARKADEAHQADIRKLEDELKGIE
AKMKTFEDAIAGESKKRGSNVHLEQNLVQEYDRLKQKADATSGKYLIKLDSVNREQKSDQDLLDSEINKK
AQIEENYKKYESERNEAAKRQEKLIDHIKASKQALEEQNRLKQELSQDVGSSKERILELQAELDDVREQL
GDAKIDKHEDARRKKKQEVVELFKQEVPGVYDRMINMCQPTHKRYNVAVTKVLGKYMEAIIVDTEKTARR
CIQILKEKMLDVETFLPLDYLQKKPLKERLRNIEDPRNVKLIYDVLKFSPPQIEPAVLFATNNALVCETP
DDAMKVAYEIDRSRYDALALDGTFYQKSGIISGGSHDLARKAKRWDEKHMAQLKAQKEKITEELKEVMKK
TRRQGELTTVESQIRGLENRLKYSQNDLDASKKNIKEYDKKLAALQLELDQIGPKISEIERRMQQRDLKI
QEIKENMNNVEDDVYAEFCARIGVANIRQFEERELVLQQERAKKRAEFEQQIDRINNNLEFERSKDTSKN
VTRWERAVQDDEDSLETFKQAEARQRQEIEKDKERIEKMKVDKAAKKAAVDAMEEEMAKARRDVQQQAKE
LAAVHQQIAGIESKIETMKNKRHNLLMQCKMDAIEIPMKRGKMNDIVEQTGGNESETTPLSTIYEREAKI
EIDYSSLPKNLTNPSEPDQVKKVGDGLARELQSKLDTLEKIQTPNLKAMQKLDRVTEKIQTTNEEFEAAR
KKAKKAKAAFEKIKNERCTLFTNCCNHISDAIDGIYKQLARNEAAQAYLGPDNPEEPYLDGINYNCVAPG
KRFQPMSNLSGGEKTIAALALLFAIHSFQPAPFFVLDEIDAALDNTNIGKVASYIREKCTNLQTVVISLK
EEFYSHADILIGICPEPAECLVSQTLIYDLEQFTPHS

>AAEL015592-PA

MKVAYEIDRSRYDALALDGTFYQKSGIISGGSHDLARKAKRWDEKHMAQLKAQKEKITEELKEVMKKTRR
QGELTTVESQIRGLENRLKYSQNDLDASKKNIKEYDKKLAALQLELDQIGPKISEIERRMQQRDLKIQEI
KENMNNVEDDVYAEFCARIGVANIRQFEERELVLQQERAKKRAEFEQQIDRINNNLEFERSKDTSKNVTR
WERAVQDDEDSLETFKQAEARQRQEIEKDKERIEKMKVDKAAKKAAVDAMEEEMAKARRDVQQQAKELAA
VHQQIAGIESKIETMKNKRHNLLMQCKMDAIEIPMKRGKMNDIVEQTGGNESETTPLSTIYEREAKIEID
YSSLPKNLTNPSEPDQVKKVGDGLARELQSKLDTLEKIQTPNLKAMQKLDRVTEKIQTTNEEFEAARKKA
KKAKAAFEKIKNERCTLFTNCCNHISDAIDGIYKQLARNEAAQAYLGPDNPEEPYLDGINYNCVAPGKRF
QPMSNLSGGEKTIAALALLFAIHSFQPAPFFVLDEIDAALDNTNIGKVASYIREKCTNLQTVVISLKEEF
YSHADILIGICPEPAECLVSQTLIYDLEQFTPHS

>AAEL003449-PA.NBD1
MYIKSIIIDGFKSYGKRTEIHGFDPEFNAITGLNGTGKSNILDSICFVLGISNLVHVRATSLQELVYKSG
QAGVTKATVTLVFDNTDKDQCPLGYEKCNEISITRQIVVGGKNKYLINGKTVQNKKVQDLFCSVQLNVNN
PNFLIMQGRITKVLNMKPQEILSMIEEAAGTSVYEAKREHSIKLIEKKDAKLNELYTVLREEIEPKLEKL
RKERAHYIEYQKICRDIEYLTRLYISHKYLQHVKSVENSEKAIAALNDVISGHRNKIEAHITESKEIEQS
AKALQELIDTEGGGALAELEAELSAESKKEATASAERDSTKENIGAEQRKLKNLQKSIRDDENALQKKEA
EMQRTGDMFQSLKDADEADAKAFTDAQKRFEAVSAGLSTNEDGEAATLQDQLMSAKQKAAESATSIKQSE
MELKHCQQLLKEKQGDMNSSDAAYLEDKKKLTKAESQIQSITAELQKIDYEEGSIEQLHERKQVLSHEIR
NLKSELDRKNAYRWEFQYRDPEPNFDRSSVHGMVAKLVAVRDKKYALSLGTAAGGSLYSVVVDSDTVGKK
LLQKGQLQSRTTMIPLNKITSRMIDNNTVRVADNLVGKGRAVTALSCINYERQLEPAMKFVFGHSFLADD
MNVAKQVTYHPQIMSRSVTLDGDVVDPSGTLSGGAKAKGAVVLLEVEEINQIQKLLAQKEAEYQQVCAEI
SKLERIAHRYNQLKEQQDMMNYELKTLQQRLAQTSYQQAQQEIEELKAKIETLKQTMVDARQAQTQANAK
VKDLTAKIADSKGHRERELKAAEDELKRAKKKSEESRKNWKKHEQSFETLRLEIEELQKGIATAKEQAAK
LEETIAELQRKLQAASENSAEMNKAVAAVKQKIKEHKDKINSQNKELKAKYHHRDKLLKQNEEMELDIKK
KENEIQKVRNENKDGYNKISALEEKYPWISEDKEFFGVKNTRYDYNKEDPQEAGRKLNKLQDSKEKMSRN
INQKAMVLLEREEEQYKEVMRRKQVVEDDKKKIQKIITDLDEEKKKQLKVAWTEVNENFGSIFSTLLPGT
QAQLKPPDGVDFMKGLEVKVGFNGMWKESLTELSGGQRSLVALSLILAMLKYKPAPLYILDEVDAALDLS
HTQNIGNMLKAHFTNSQFVIVSLKDGMFNNANVLFRTKFIDGMSGVTRTVNVAHAKAKAAGR

>AAEL006937-PA.NBD1
MHIKQVIIQGFKSYREQTVVEPFDKRHNVVVGRNGSGKSNFFYAIQFVLSDEFTHLRPEQRQALLHEGTG
ARAMSAYVEIIFDNSDNRVPIDKEEIFLRRVIGAKKDQYFLNKKVVPRSEVVNLLESAGFSNSNPYYIVK
QGKINQMATAPDSHRLKLLREVAGTRVYDERKEESMNILKETDGKVEKITEFLKTIEDRLKTLEEEKEEL
KEYQKWDKARRTLEYIIHETELKENKKHLDELEHQRKSSGDKQKMFAQEIQTAQDKIKNAQKALKDAKKE
VTTTKDEKSVLTTEHQQLLREKTKLDLTISDLTDEVQGDNKSKERAEQELNRLKLTIAEKEKELEEVRPR
YEAMRRKEEECSRELNLKEQKRKELYAKQGRGSQFSSREERDKWIQGELKSLNKQIKDKIAHQNKLQEDL
KRDAAKQKDLEKKIEEHTESFEQLRIQIDEHNKHYYELKKKKDHFQTIRNDIWKKETQVTQTLSGNKEEL
AKADQALRSMAGKPILNGRDSVRKVLESFSQRGGQFADIARSYYGPVIENFNCDKSIYTAVEVTAGNRLF
HHIVESDRVGTQILKEMNKQKLPGEVTFMPLNRLQVRIHDYPEDPDSIPMISKLKYEEQYDKALRYIFGK
TLICRNLERATELAKSTGLDCVTLEGDQVSSKGCLTGGYFNTSRSRLEMQKKRSEYLQMIEDLEKELSEF
RTELKQTESSINSVVSEMQKTETKQGKSKDAFEKIQADIRLMKDELTRIERFRNPKERSLAQCKANLEAM
NSTKDGLENELHQELMSQLSVQDQHEVDQLNDEIRKLNQENKEAFTARMSLEVTKNKLENLLTNNLFRRK
DELVQALQEISVEDRKRQLTNCRTELTSTEKRIKKVLSDVDDIDKKVSEAVKQQKALQKELELWMQKEKE
AQDKMEEDSKRMEKWAAKENLLHQKIEECTEKIASLGALPQVDPSYQRMSLKTLFKELEKANHHLKKYNH
VNKKALDQFLSFSEQKEKLYKRKEELDIGGEKIRELMQNLEMRKVEAIQFTFRQVAANFTEVFKKLVPQG
SGHLILRTQKDTEGNDMEKEVETSDEFTGIGIRVSFTGVDAEMREMNQLSGGQKSLVALALIFAIQKCDP
APFYLFDEIDQALDAQHRKAVADMIHELSDKAQFITTTFRPELLENAHKFYGVRFRNKVSHVDCVTREIA
RDFVEDDTTHG

>AAEL001655-PA.NBD1
MSKNKRRSGGAAGQPPAAAQQPPAEAMEVDIDDDANISEDEEGGTRVGGIYIPPPVPPYCSTECKGPRLI
ITHISNYNFKSYAGHVMLGPFHQRFSAIIGPNGSGKSNVIDSMLFVFGYRAQKIRSKKLSVLLHSSSKHP
NTNSCSVGVHFKQIIDKEDGSFESVPNSEFVVARTAFRDNSSYYTVDNKRVHFKEVSKLLKQHGIDLDHN
RFLILQGEVESISMMKPKAMTENDCGLLEYLEDIVGTTRYKQPLVKINERVELLNEERTEKHNRCKLAER
EMKDLEKPMHEAVEYLKLENSLTRTKNQQIQKYISEQLKKITELEVEREQAGGILKKHDETYEALKKERV
EKEHLVKEEIKQYDGLVKKKDDKEAALKASLANYTKVQANMKATNERRKKTKEQIEAEKKKLLDLKEIPE
KNAKEIAESEKKIESLTKQKGDIEAKLTENLATLKDETKVLLEEKEKLQTELIDLKKAVDDSKSALSLAE
SELKICQHNETTEKRKLETLKYSFEDSNKNFQEKQARLQELEKNLPQLRTDLQTAKAKLQENVTEEKELR
SQLRTVQGKLQESMQAVQSTRSQGKVLDALMKQKNEGRIPGILGRLGNLGGIDSKFDVAISTCCGHLDFI
VVETVNTAQACIDYLKKSDIGRASFIALEKIQQYQQYCHRQIQTPENVPRLFDLIHVEDERVLPAFYFAL
RDTLVAENLDQGQRIAYGAKRYRVVTLGGDVIETSGTMSGGGRSMQRGRMGTKVQTKTSASDTPKSNKEL
EHLQVRAQEIQSQINFLQEQQGELEQQIQRMGSQLKNQENEVKRLKMDVTSLAEQLPRLKDQVERQQARV
AQTHSDPEKVRALEAQVAECKETHTKSSEAANTIQKQVDGVTKKINDITNSKVKDLQTKINNLTKQIDKL
TTNISKLTVEVKTSERNVKKTEDKIKNMEEEVISAEQAIRSGCEEREALEKEASDLKQEIDELKVEIEKA
HDGSSSIKKEIVAIQKKEAEGKMKRLEFEQILQSIEKKLSEVKDTIPHWKNKLKPLKLHEIPGEPAPEPL
KEYTEEELNSYKLSDLQYQFSIQEEKLNASKPNLSVIEEFLRKQEAYLQRVAILEEITAKRNEMRQLYDD
VRKKRFTEFMQGFHIITKKLKEMYQMITLGGDAELELVDSMDPFNEGIVFSVRPPKKSWKMISNLSGGEK
TLSSLALVFALHYYKPSPLYVMDEIDAALDFKNVSIVAHYIKERTKNAQFVIISLRSNMFELSDYLVGIY
KVTDCTNSVTIENDPPKFHHSSTQQQSQSFFNPNLSLQSDLGLDQSLPKDEKTKTEDSLQKSDSQKENIP
EPSPVSQQPMEVSVEAN

>AAEL002581-PA.NBD1
MGHVFEDGPAPTRKRYCINSASIEFMPAGSERKDHKLKTKHTGSKSSQQVQIVAPQADKVERAIIHDIEK
YVRSGKILKMQLKNFMCHRNLVVEFNKRANLLVGNNGSGKSAVLAALTIGLGCSANLTNRSSSVKQLIKH
GETQASIEIHLGNDSFDAYERDVYGDRIIIIRTINASGATSYKLKSEQGRVISQSRSDLLKMILFLNIQV
DNPVCVLNQDLARSFLKDSDEKKQYNLFLKATQIDSITAKLNGCTPQLENAKHNLDCNEKSLKYIEREIE
EMQQKYENLQSVEKWKDQVKLARLKLGWRVVIDQFTECSQVEDKLREKVNTMKEHMNAIQNREALEAEID
RVIQRFRNDIDAKKTGFAEVKDKYMQARRVGQSLQEQLSDKSRQMKKVKDRLARQAEDIKNLEADLKQRS
ESGSNRMADEKRRNEQQLSELSEKKRDLQAMVENAKRDVDILHNTLNQIRETKDEANSKRVSKQHERTRT
EMQLQQFESSSRDNLSVFGQNMPAFVAKIKQMHQQGRFTELPRGPLGQYIKVKDKKWTAMIETVISPGIL
TAFYVNSDADRNTLNQLIQREFPEMRGRSIITSRFHKEVYDVRSGRVETVQNAHMLMNLINVSDPVVMNC
LIDQIKIETILVVDDQNLAMDLTSHTENVPKNLQKVVVMEPFSEFFPMPNYRSYGLQKKSARYLQVNMTE
LKKQTERRMQQLDQELNEIKHQIEEETRKLGERERALQDKQRQMAKQQQELNSLELKINELKAIEYPAES
EDMTLQNELEELKASQIKLNEELEQEKSKLQSSVNEITDQERIIQEKKDHMTKIESEIQEIQDKIDAEMQ
KRHDMQANAKTKQQQLNRLQEEVQTMQAERNERKQALAAATSAAQELGERVEVKETQEQLKKAISSTEKR
IKNINSANDDIVDVKAILENKILKRDTSLRYTDSLKKIIKLLTNSRSARYAYLHKLKSHMSLRVKHKFNM
VMQLRGFVGEIVMDTKNGTLSLSVVPRDKNISNAVCNTKSLSGGERSYSTVAFLIALWSCVDTPFYFLDE
YDVFTDQVNRHMMTMLLLNETKKRPDRQFCFLTPQDMSNIQASDDLTIHRMADPERC

>AAEL005245-PA.NBD1
MPPNKCRMINTKEDQQNQIRSKIQNLFKGSFHELEIEIQNFKQSKSSKLLELREAESDLSSRKAQEKVLQ

SKIQDFESRRIHLNTKRGQEQDLCGDRSKKMVALCDRLKLPAVGDFEAGGADVESALKAIKHGIRSEETA
VQTLSKSHDEADYKAQKAIDKLREDKTKLEADFRMKGQMVADFVREKAKTQSEIATIQRSAETLKKIVTE
IEKLEKDYENQKSNSNVEGMRRQLAEKKIKHEELQAKLDRVEEQISALDAVAAKATELHLKEQQFNAKES
EFKRLRNKHTENLKRLFPSKTIDTNYKRNVQTLYDDLQRQIKQLNESMRKSQATVTEMETTRRSQKRELD
RIERELTENKEKIYTVCQGNPFEEVLAKLKDKISKNNLEHGELRSAEVLYKKYISRIEDDKCCPVCHKGM
DGADVQDISSELSDEIRRLPEKIEALERMLKADQKKYDQLLALQPYSERIAKQTEEIPKLKEQLEETERK
LVQASSDLEECQMSILEPNSSVQLINSILGDMSILDESVRDIDRMKKGIENLKIEVAVKTPEGTTASLDD
IKLEREALRGELRVERISIDAMQNKIDEETERLNNLHQRYNQMKEKKIQLQESVQSLDQKKAKELELGEK
IASCQREMDETENKLGPVKQNLITEEKSKAKAKEENRANLNKAQAELEELKRMDAEIVRLCKELDALSRL
NLAEEIQKIKRKLQESNEEMKKIIAAIDEKTYQIDSLKKEVMNQDMIERDYLDNRDLMKLKDETTSLKQG
LDALMQSIGDMDAPNVAQERNKLLEQRDAIQSTKSQTTGQIAELENQSKALKKELDRSEFKNAVKNYLKT
YSESVVLKKMISDILKYRNALEWALMKYHAEKMEQINRSIYSLWRDIYRGNDIDYIRIKTEDESKPDKVT
DKRRQYHYRVVQAKNDVEIDMRGRCSAGQKVLASLIIRMALAETFSNNCGVMALDEPTTNLDRENIASLC
ESLRRIVTERENGNFLLVIITHDEDFVTKLEKFDTYYRVSRDHDGKSIIKEEQL

>AAEL014748-PA.NBD1
MPCLCGMIKIETLRSTYVNSERFRWINTKEDQQNQIRSKIQNLFKGSFHELEIEIQNFKQSKSSKLLELR
EAESDLSSRKAQEKVLQSKIQDFESRRIHLNTKRGQEQDLCGDRSKKMVALCDRLKLPAVGDFEAGGADV
ESALKAIKHGIRSEETAVQTLSKSHDEADHKAQKAIDKLREDKTKLEADFRMKGQMVADFVREKAKTQSE
IAAIQRSAETLKKIVTEIEKLEKDYENQKSNSNVEGMRRQLAEKKIKREELQAKLDRVEEQISALDAVAA
KATELHLKEQQFNAKELEFKRLRNKHTENLKRLFPSKTIDTNYKRNVQTLYDDLQRQIKQLNESMRKSQA
TVTEMETTRRSQKRELDRVERELTENKEKIYTVCQGNPFEEVLAKLKDKISKNNLEHGELRSAEVLYKKY
ISRIEDDKCCPVCHKGMDGADVQDISSELSDEIRRLPEKIEALERMLKADQKKYDQLLALQPYSERIAKQ
TEEIPKLKEQLEETERKLVQASSDLEECQMSILEPNSSVQLINSILGDMSILDESVRDIDRMKKGIENLK
IEVAVKTPEGTTASLDDIKLEREALRGELRVERISIDAMQNKIDEETERLNNLHQRYNQMKEKKIQLQES
VQSLDQKKAKELELGEKIASCQREMDETENKLGPVKQNLITEEKSKAKAKEGNRANLNKAQAELEELKRM
DAEIVRLCKELDALSRLNLAEEIQKIKRKLQESNEEMKKIIAAIDEKTYQIDSLKKEVMNQDMIERDYLD
NRDLMKLKDETTSLKQGLDALMQSIGDMDAPNVAQERNKLLEQRDAIQSTKSQTTGQIAELENQSKALKK
ELDRSEFKNAVKNYLKTYSESVVLKKMISDILKYRNALEWALMKYHAEKMEQINRSIYSLWRDIYRGNDI
DYIRIKTEDESKPDKVTDKRRQYHYRVVQAKNDVEIDMRGRCSAGQKVLASLIIRMALAETFSNNCGVMA
LDEPTTNLDRENIASLCESLRRIVTERENGNFLLVIITHDEDFVTKLEKFDTYYRVSRDHDGKSIIKEEQ
L

>AAEL011780-PA.NBD
MSKNKDKLNSSNTLFNYFAKSPATPKIKPVSGSSNPGTPVSSSNGGTPKGVIKQEKVTPSPAAKDKPLNG
NDDEDEDEIQPMKKRRRIVMMDDEDGDSDSENRQKNDKTPPKAELLASFKRVEKDEASESPVQKKVKKEA
EDVAEGKPDLADLGAEEGASVLDEPTVWAHQKLDFLKPEKIKDIQGNRPSSEKYDSRTLYVPESFLGTLT
PAMRQWWELKCRHMDCVLFFKVGKFYELYHMDATVGVEELGFSYMKGEFAHSGFPEQAYERMATLLVDKG
FKVARVEQTETPDMMQERCRKNKTNSKYDKVVKREICQVSLKGTEVFGQQVHLTLNHQPKYMLAITERIK
GEVGSRYGVCFIDTSLGVFHVGEFEDDSQGSRLLTLLSHYAPALVLQERNVVSAATQQIFKTVLAGIRKE
ALTNESQFWSAERTLKYLAENFYGGSTDDQNSKWPEVIRSLLDKSDHLGLTPDGNFKLALKALGGCIWYL
KRCLLDQQIISLASFVMYIPPDDVETCKNLRIVNSNRFMVLDSVTLNNLRITEGEQSLVNRMDHCCTKFG
KRLLHHWICSPSCERDVIVQRQEAVTELIEDVSLLQDVRQILGELPDLERMLAQIHTFGNAERSKNHPDG
RAILYEEQTYGKKKIQDFINTLKGFGTLTKLPELFGKVSSKLLVQLTQTSERGGSFPDMADKIRFFDEAF
DHEQALKDGVIAPGEGLDAEYDAVHKEIKNILEELEEYKRKQEKYFGCKIAFFGTDKKRFQLEIPESAAK
KANSGYTLEGHKKGKNGVKRYHTDETREFLKRMMQTEDQRKMVLKDLSRRIFEKFSSSYEMWRTCIDLTG
TLDVLTSLAEYARSEGNMCVPEILDDSAGQVFELEEGIHPCVSDSENYIPNGVNIGGEGAPLVLLTGPNI
GGKSTMMRQVGILAVMAQIGSRIPAESCRMTLIDRIFTRLGANDDIMAGHSTFLVELNETSTILKHATRK
SLVLLDELGRGTATYDGTSIAGAVVNFLADLKCRSMFSTHYHNLVDNFSTDSRVRLGHMACMVENEDDED
PTQETVTFLYKYADGACPKSYGFNAAKLAGMPACIIKRAHELSKTVEADALKRRIFTNSLLQEDPHDLKE
LLNKLKACRF
